# Identifying a real space measure of charge-shift bonding with probability density analysis†

**DOI:** 10.1039/d4sc01674b

**Published:** 2024-05-13

**Authors:** Michel V. Heinz, Leonard Reuter, Arne Lüchow

**Affiliations:** a Institute of Physical Chemistry, RWTH Aachen University Landoltweg 2 52074 Aachen Germany luechow@pc.rwth-aachen.de +49 241 80 94748

## Abstract

Charge-shift bonds have been hypothesized as a third type of chemical bonds in addition to covalent and ionic bonds. They have first been described with valence bond theory where they are identified by the resonance energy resulting from ionic contributions. While other indicators have been described, a clear real space fingerprint for charge-shift bonding is still lacking. Probability density analysis has been developed as a real space method, allowing chemical bonding to be identified from the many-electron probability density |*Ψ*|^2^ where the wave function *Ψ* can be obtained from any quantum chemical method. Recently, barriers of a probability potential, which depends on this density, have proven to be good measures for delocalization and covalent bonding. In this work, we employ many examples to demonstrate that a well-suited measure for charge-shift bonding can be defined within the framework of probability density analysis. This measure correlates well with the charge-shift resonance energy from valence bond theory and thus strongly supports the charge-shift bonding concept. It is, unlike the charge-shift resonance energy, not dependent on a reference state. Moreover, it is independent of the polarity of the bond, suggesting to characterize bonds in molecules by both their polarity and their charge-shift character.

## Abstract 2

Charge-shift bonding is discovered independently of valence bond theory with probability density analysis. A shift of charge facilitates the sharing of electrons and increases delocalization.

## Graphical abstract

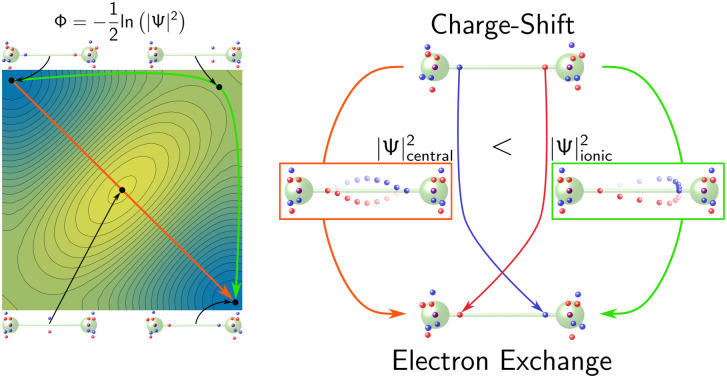

Traditionally, there are but two categories for bonds in molecules: ionic and covalent.^1^ With the quantum mechanical framework of valence bond (VB) theory, Pauling replaced this discrete dual picture with a continuous one. Within VB theory, any wave function describing a two-center two-electron bond is constructed as a linear combination of one covalent and two ionic resonance structures (Fig. 1).^2^ Still, the covalent-ionic duality dominated the discourse and while the continuous definition improves every wave function quantitatively, it rarely changes the picture qualitatively: for the C–C bond in ethane, the covalent wave function alone already leads to a marked minimum in the dissociation energy curve (Fig. 2a). Yet, for other systems (*e.g.* F_2_), the covalent wave function alone leads to qualitatively incorrect results, since the resonance between the covalent and the ionic structures is essential for the description of the bond (Fig. 2b).

**Fig. 1:**
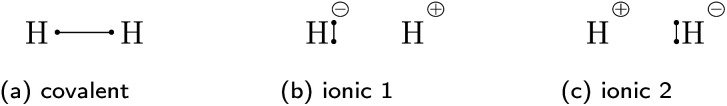
The three valence bond resonance structures of two-center two-electron bonds shown for the hydrogen molecule.

**Fig. 2:**
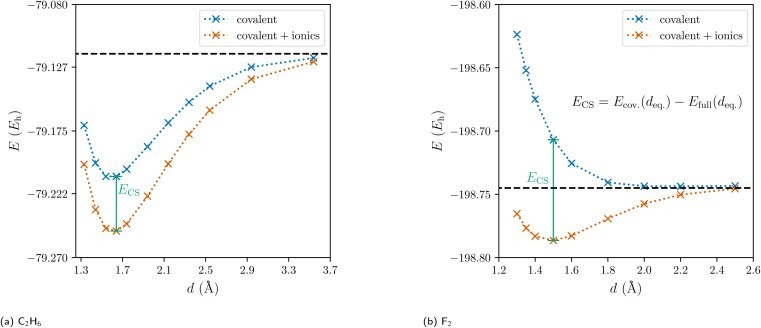
Dissociation energy curves of the covalent wave function (blue) and of the full valence bond wave function (orange) for ethane and the fluorine molecule.

Since the ionic structures can be constructed from the covalent structure by shifting a charge, Shaik and coworkers coined the term ‘charge-shift bond’ for these latter systems and thereby added a third category of bonds to the continuous picture.^3–6^ For valence bond theory, they established the charge-shift resonance energy *E*_CS_ to quantify a bond's charge-shift character. With the introduction of an ionic charge-shift resonance energy for predominantly ionic bonds, they generalized this measure:1
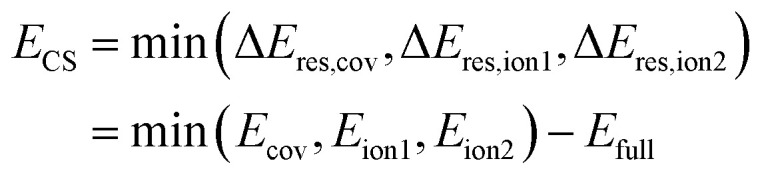


The charge-shift resonance energy has been shown to have great predictive capability for the comparison of barriers of hydrogen abstraction and halogen exchange.^7^

There have already been several approaches to identify charge-shift bonds outside of VB theory. A real space measure based on a positive Laplacian of the electron density at the bond critical point has been proposed by Zhang *et al.*^8^ Yet, while the sign of the Laplacian works as an indicator to distinguish the classic examples F_2_ and C_2_H_6_, a good correlation with the charge-shift resonance energy in general is only obtained for the covalent and the resonance contributions, the calculation of which again requires valence bond theory. Another proposed indicator is the ratio of the population and the population-variance within basins of the electron localization function.^5^ This ratio is between 1.0 and 1.8 for charge-shift bonds, while it is usually larger than 2.0 for covalent or ionic bonds. Still, the usefulness of the charge-shift concept outside of VB theory is debated.^9^

In this work, the recently developed probability density analysis (PDA) is used to identify charge-shift bonds in many-electron coordinate space. PDA is the many-electron analogue of the quantum theory of atoms in molecules (QTAIM) by Bader and coworkers.^10–12^ It is based on the analysis of the critical points and a topological analysis of the many-electron probability density |*Ψ*|^2^ instead of the electron density, where *Ψ* is the many-electron wave function. While local maxima of the electron density (nuclear critical points, NCP) are almost exclusively found at the nuclei, the maxima of |*Ψ*|^2^ are the locally most probable arrangements of all electrons simultaneously. These maxima, denoted structure critical points (SCP), correspond in many cases directly to the familiar Lewis structures.^11,13–15^ In QTAIM, the basins of attraction around NCPs are denoted quantum atoms. In PDA, the basins around the SCPs allow the partitioning of the many-electron probability density into contributions of different electron arrangements. Due to the indistinguishability of electrons and the large number of possible spin permutations, many equivalent or similar SCPs are identified. These similar SCPs are subsequently clustered into PDA structures.^11,16^ A weight for each PDA structure is obtained by integration over the respective union of SCP basins. Not only do the PDA structures usually correspond to VB resonance structures, but also the PDA structure weights have been shown to be in good agreement with VB weights.^12,17^ Note that PDA works for arbitrary many-electron wave functions, thus allowing to extract VB-type resonance structures from MO-based wave functions.

Covalent bonding is usually understood as being caused by electron sharing between atoms, requiring a delocalization of electrons.^18^ In the simplest case, H_2_^+^, the delocalization is characterized by the saddle point of |*Ψ*|^2^ in between the two protons. In contrast to the bonding ground state ^2^Σ_g_^+^, the probability density vanishes between the nuclei for the antibonding first excited state ^2^Σ_u_+, indicating diminished delocalization and thus antibonding (Fig. 3). Since QTAIM and PDA are evidently equivalent for one-electron systems like H_2_^+^, this saddle point of the electron density is a bond critical point (BCP). Yet, in PDA in general the saddle points characterize the delocalization of electrons and are thus termed delocalization critical points (DCPs). Saddle points of higher order are denoted as higher order DCPs. While in QTAIM BCPs connect two atoms *via* a bond path, first-order DCPs connect two SCPs, *i.e.* probable electron arrangements.^19^

**Fig. 3:**
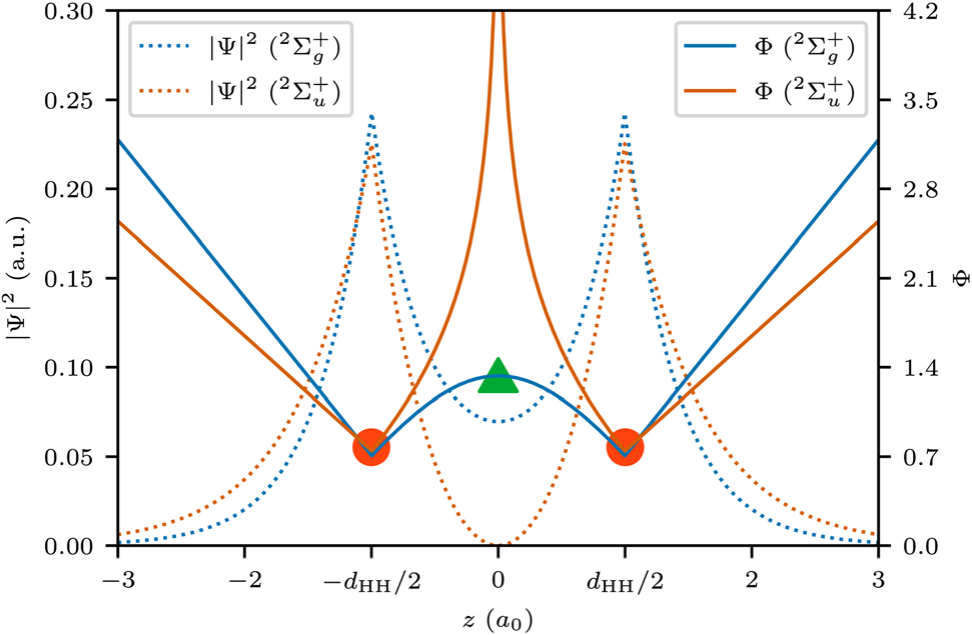
Probability density |*Ψ*|^2^ (dashed lines) and probability potential *Φ* (solid lines) along the bond axis *z* for H_2_^+^. SCPs and DCP are indicated for the ground state probability potential.

In addition to the discussion of these critical points, any path in many-electron coordinate space can be analyzed. Paths describing the exchange of electrons are intricately linked to chemical bonding. Since independent probabilities (for independent bonds in a molecule) multiply, a probability potential2
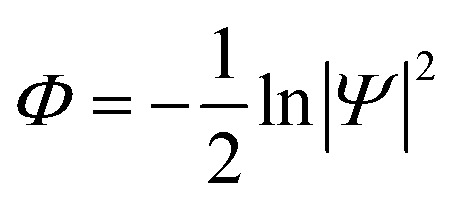
is defined within PDA.^19^ Independent parts of a molecule add a constant which vanishes in differences of the probability potential. Furthermore, since the sign is reversed, the SCPs are now local minima of *Φ* and the DCPs have larger potential values than the neighboring SCPs.

With this probability potential, a dimensionless probability barrier can be defined for arbitrary paths in many-electron coordinate space as the difference between the maximal probability potential *Φ*_max_ along the path and the starting probability potential *Φ*_start_:

3 Δ*Φ* = *Φ*_max_ − *Φ*_start_

An ordinary covalent single bond is characterized by a first-order DCP, describing the exchange of the two bond electrons. The barrier for the lowest path between the two neighboring SCPs is readily identified as:

4 Δ*Φ* = *Φ*_DCP_ − *Φ*_SCP_

The DCP connecting two SCPs is best understood as the analogue of a transition state connecting reactant and product in a chemical reaction. Accordingly, the barrier Δ*Φ* is the analogue to the activation energy (see Fig. 3). The lower this barrier the stronger the delocalization between two SCPs. The path connecting two neighboring SCPs *via* a first-order DCP is termed delocalization path. This path is 3*n* dimensional and describes a movement of all *n* electrons. For H_2_^+^, the delocalization path is the straight line connecting the protons. For larger molecules delocalization paths can describe localized two-electron exchanges but also many-electron processes. Yet, delocalization paths do not correspond to observable electronic processes in molecules.

## Results and discussion

### Homoatomic bonds

**Fig. 4:**
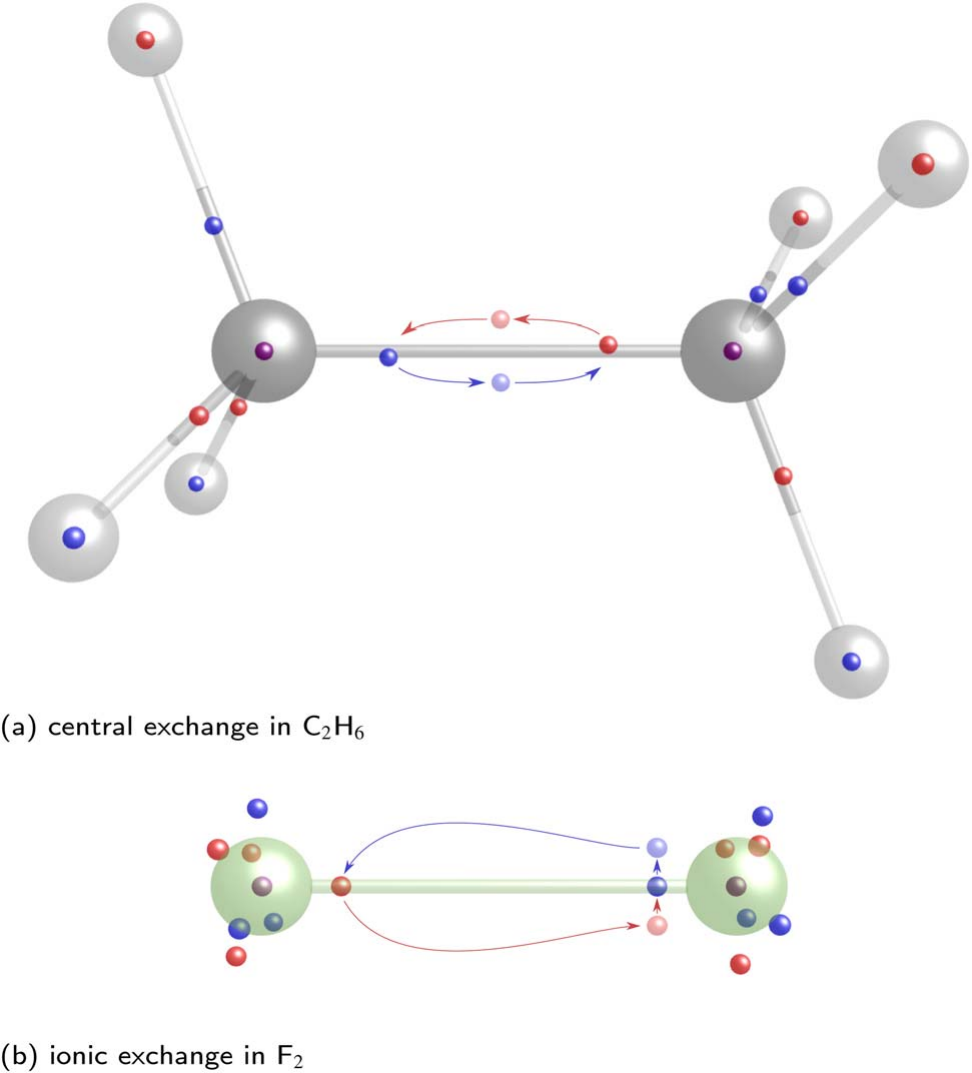
Central exchange path for C_2_H_6_ (a) and ionic exchange path for F_2_ (b) for explicitly-correlated multi-reference wave functions. Nuclei positions are depicted by grey, dark grey and green spheres for hydrogen, carbon, and fluorine respectively. Electron positions of the starting SCP are depicted by smaller red and blue spheres with the colors indicating the secondary spin quantum numbers. The delocalization paths are depicted with arrows with the electron positions at the DCP being displayed as brighter spheres.

In previous work, the SCPs, PDA structures, and weights of homoatomic bonds have been discussed in detail,^11,12,16^ while DCPs and barriers have only been identified and calculated for H_2_ and H_2_^+^.^19^ For this work, we started by investigating explicitly correlated multi-reference wave functions of the homoatomic bonds between first-row atoms in C_2_H_6_, N_2_H_4_, H_2_O_2_, and F_2_. The identified SCPs again resemble Lewis dot structures (Fig. 4). While the delocalization path for the central two-electron exchange in ethane describes a concerted exchange *via* a symmetric DCP (Fig. 4a), the exchange in F_2_ passes an ionic DCP (Fig. 4b). These are first-order DCPs—delocalization paths can never pass higher order DCPs. The obvious difference between both homoatomic bonds is therefore the ionic character of the F_2_ DCP compared to the central non-ionic exchange in C_2_H_6_. While the C–C and the F–F bond are both described by a two-electron exchange delocalization, the F–F delocalization is marked by a strong “charge shift”.

Since correlated wave functions are employed, the opposite spin electrons are found at a distance in all SCPs and DCPs. Additionally, low-barrier DCPs exchanging two electrons of any of the C–H bonds are identified for C_2_H_6_, while many DCPs involving the lone pair electrons are identified in F_2_ with even lower, almost negligible barriers. Yet, these DCPs do not concern the central homoatomic bond in both molecules.

In order to visualize more of the topology of the probability potential for different two-center two-electron bonds, a reduced two-dimensional potential is constructed. The molecule is reoriented such that the bond of interest lies on the *z* axis of the coordinate system and the center of the bond is at *z* = 0. The value of the reduced potential can then be calculated for any point (*z*_1_, *z*_2_) in two steps:

(1) The *z* coordinates of two arbitrary electrons are set to *z*_1_ and *z*_2_. These are hereafter denoted as the bond electrons.

(2) Subsequently, all other coordinates (*i.e. x* and *y* coordinates of the bond electrons as well as *x*, *y*, and *z* coordinates of all other electrons) are optimized by minimizing *Φ*. The resulting minimal value of *Φ* is the value of the reduced potential.

**Fig. 5:**
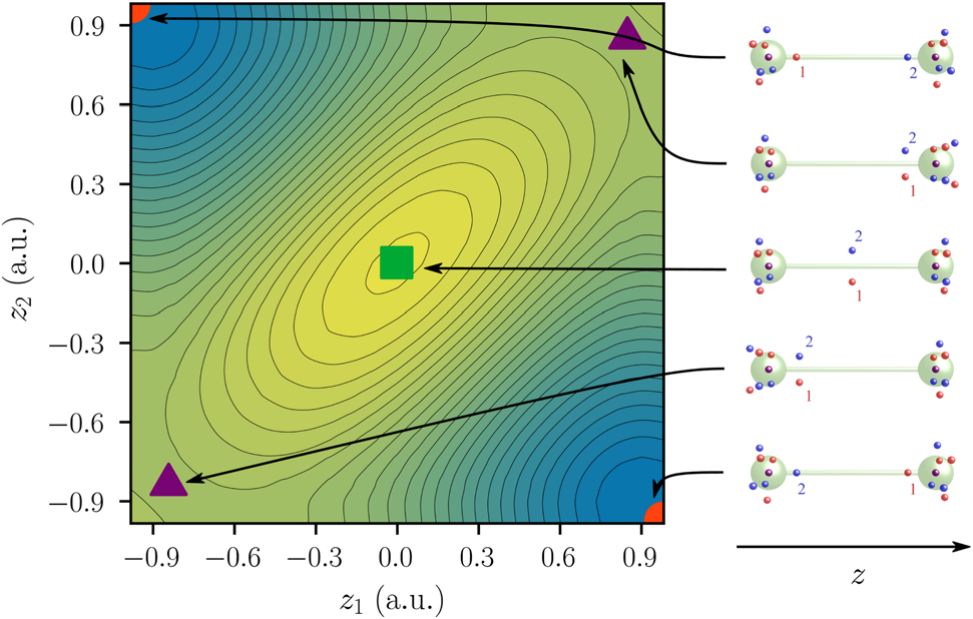
Two-electron probability potential of F_2_ centered at *z* = 0. SCPs are depicted as circles, first-order DCPs as triangles, and second-order DCPs as squares. Covalent arrangements, ionic arrangements, and the central arrangement are depicted in red, violet, and green, respectively. The respective electron arrangements are shown on the right. The electron indices 1 and 2 on the right hand side correspond to the two axes *z*_1_ and *z*_2_.

This way, the critical points (SCPs and DCPs) appear in the reduced potential. For F_2_, five critical points—two SCPs, two first-order DCPs and one second-order DCP—are identified within this two-dimensional potential (Fig. 5).

The color scale of the reduced potential ranges from blue (low values) to yellow (large values).

**Fig. 6:**
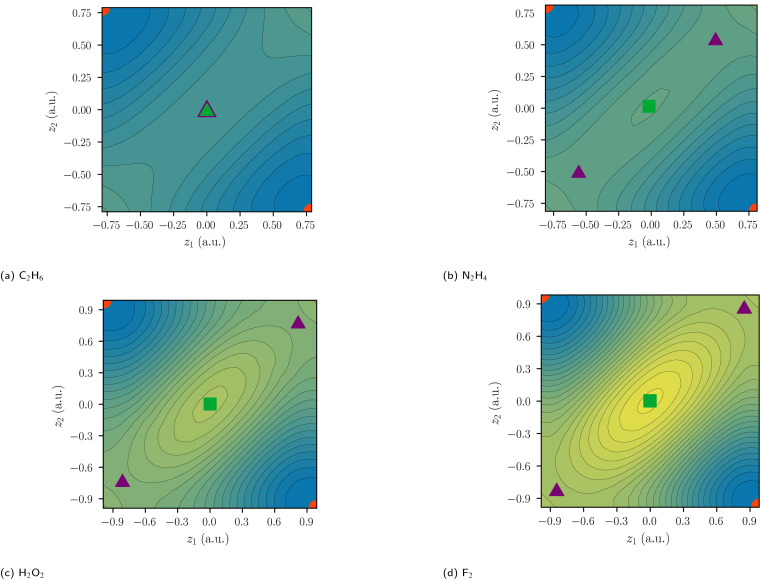
Two-electron probability potentials of homoatomic bonds centered at *z* = 0. SCPs are depicted as circles, first-order DCPs as triangles, and second-order DCPs as squares. Covalent arrangements, ionic arrangements, and central arrangements are depicted in red, violet, and green, respectively. The color scale of the reduced potential ranges from blue (low values) to yellow (large values).

While there are similarly five critical points for N_2_H_4_ and H_2_O_2_, only three critical points are identified for C_2_H_6_ (Fig. 6). For N_2_H_4_, H_2_O_2_, and F_2_, the delocalization path for the two-electron exchange on the central bond is *via* one of two equivalent ionic first-order DCPs with a lower barrier than the central exchange path *via* a second-order DCP. The difference of the probability potential barrier between the two paths increases from N_2_H_4_, H_2_O_2_, and F_2_ and with it the “charge shift” character of the bond. This difference between the central probability barrier *Δ*_C_*Φ* and the ionic barrier *Δ*_I_*Φ* is therefore proposed as a measure of charge-shift character and denoted charge-shift strength *Δ*_CS_*Φ*:

5 *Δ*_CS_*Φ* = *Δ*_C_*Φ* − *Δ*_I_*Φ*

In the series of F_2_ to C_2_H_6_ the ionic contributions and thus *Δ*_CS_*Φ* decrease up to the point, where the ionic DCPs merge with the central DCP for ethane and *Δ*_CS_*Φ* is exactly zero. Therefore, if no ionic DCP exists, as in C_2_H_6_, the charge-shift strength *Δ*_CS_*Φ* is set to zero. Thus, the definition differs qualitatively from the VB charge-shift resonance energy, which is strictly positive. Note that this real-space definition of the charge-shift character does not require a covalent reference state.

### Heteroatomic bonds

**Fig. 7:**
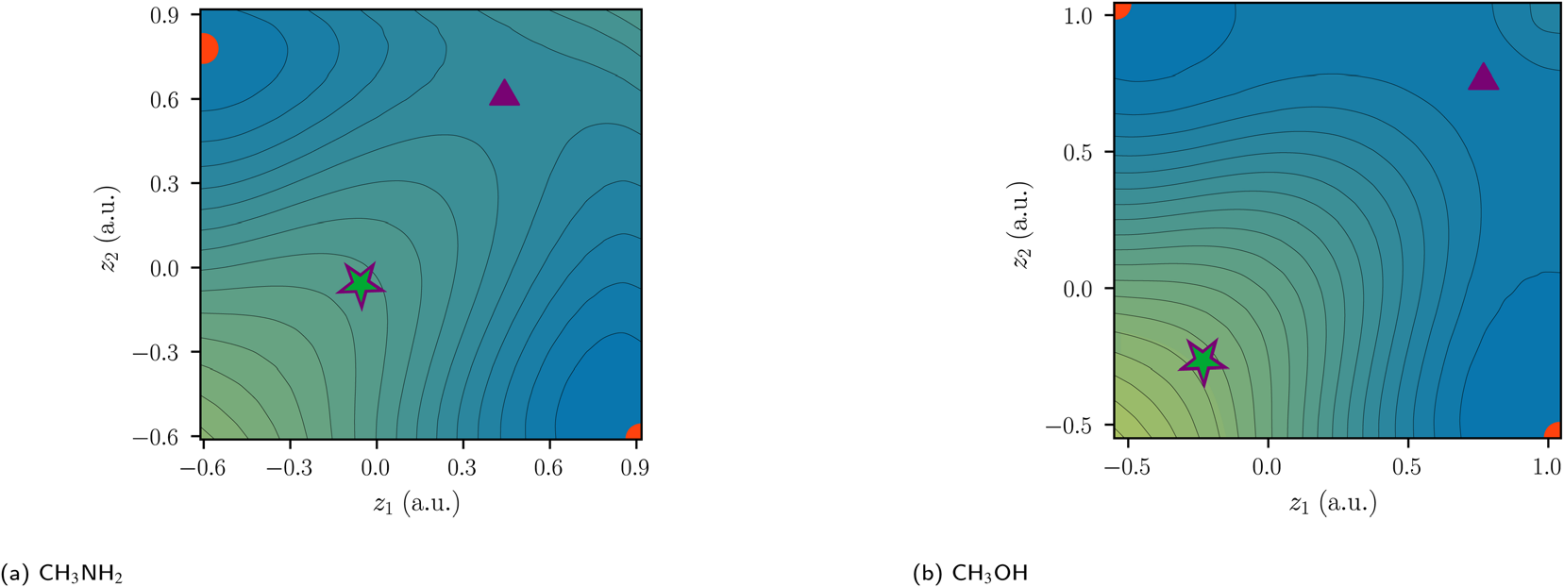
Two-coordinate potentials of heteroatomic bonds centered at *z* = 0. SCPs are depicted as circles, first-order DCPs as triangles and other arrangements as stars. Covalent arrangements, ionic arrangements, and central arrangements are depicted in red, violet, and green, respectively.

In polar heteroatomic bonds, often only one ionic path is found, and no central DCP can be identified. In the latter case, the central path is defined to include the point of locally minimal slope on the ridge between the two covalent minima. This point in coordinate space is denoted as central arrangement (Fig. 7). While it is numerically obtained by fitting polynomials on points along the ridge, the definition itself is robust and not reliant on the fitting procedure.

**Fig. 8:**
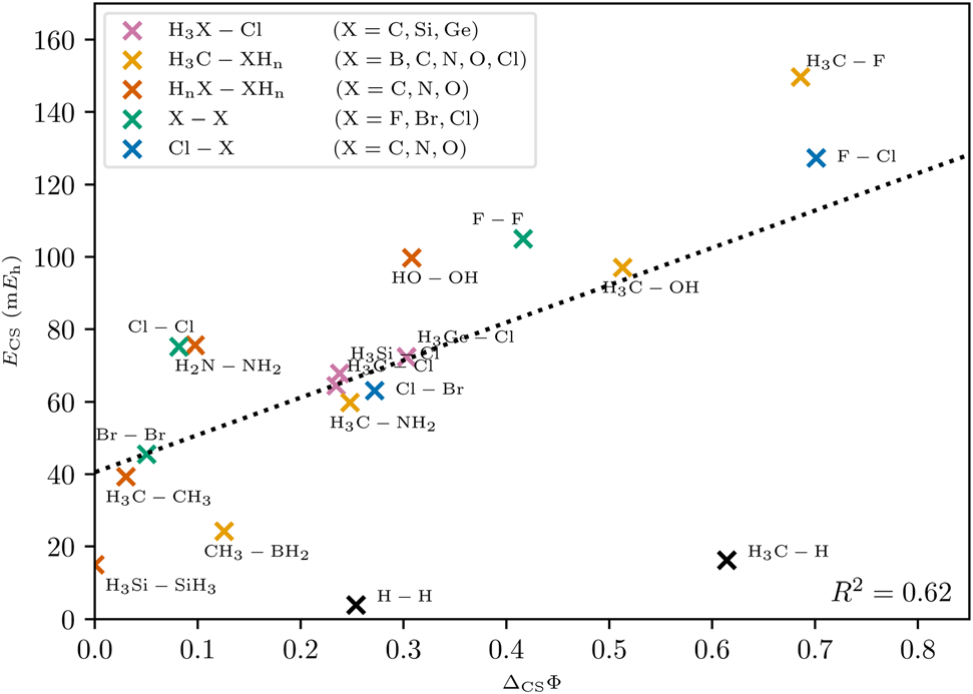
Correlation between charge-shift resonance energy of VB theory and charge-shift strength of PDA. The linear trend is depicted as a dotted line. Different homologous series are indicated with different colors. The coefficient of determination *R*^2^ and the linear regression were calculated without the outliers H_2_ and CH_4_.

With this generalized definition, the charge-shift strength *Δ*_CS_*Φ* is compared to the charge-shift resonance energy of VB theory (8) for a range of small molecules. It is expected, that the two descriptors of the charge-shift character show the same trends and identify the same bonds as charge-shift bonds. For the covalent VB wave function of F_2_, the ionic paths vanish and both *Δ*_CS_*Φ* and (by definition) *E*_CS_ are zero. Adding ionic contributions leads to an increase of both charge-shift descriptors. It can be seen that the charge-shift strength obtained with PDA is strongly correlated with the resonance energy. This correlation is even better if only the molecules of a homologous series are considered (*cf.* the different colors in Fig. 8). Therefore, the charge-shift strength is capable of describing the same effect as the charge-shift resonance energy in a quantitative way. Moreover, the *Δ*_CS_*Φ* values start at zero for the prototypical normal covalent bond in ethane. While the charge-shift resonance energy is limited to VB theory, the charge-shift strength can be calculated for any wave function.

The only outliers in this investigation are H_2_ and the C–H bond in CH_4_: while the VB charge-shift resonance energy *E*_CS_ of H_2_ is the lowest of all systems investigated, the PDA charge-shift strength *Δ*_CS_*Φ* is larger than that of the prototypical charge-shift bond in F_2_. Yet, the bond in H_2_ is already known to behave as an outlier with many other methods.^20–22^ The outlier position here probably results from the missing core electrons and the wave function cusps at the proton positions. The argument that the discrepancy lies with the hydrogen atom is supported by a similar discrepancy for the C–H bond.

### Polarity and charge-shift strength: two dimensions of bond character

While Shaik and coworkers usually present charge-shift bonds as a “third family of bonds”^6^ alongside covalent and ionic bonds, they also occasionally make a distinction between covalent charge-shift bonds and ionic charge-shift bonds (*cf.*eqn (1)).^23^ We want to build upon this distinction and establish two separate dimensions of bond character: polarity and charge-shift character.

**Fig. 9:**
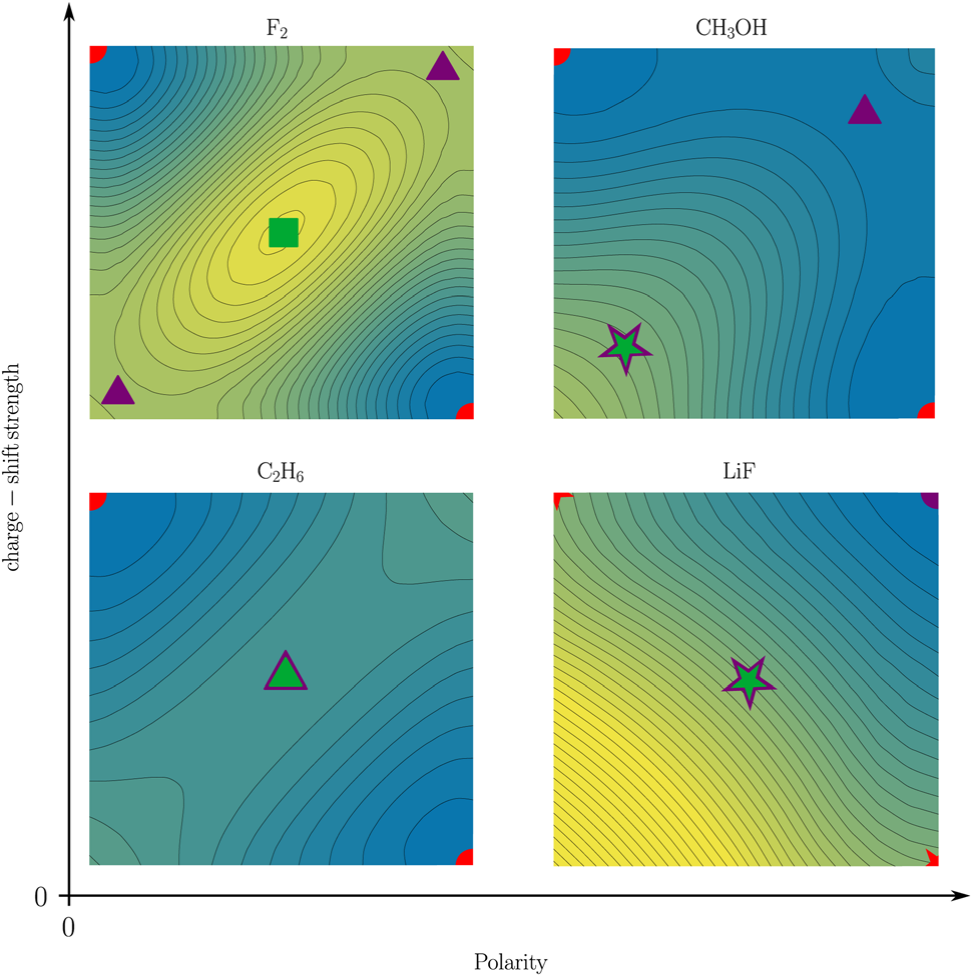
Two-coordinate potentials of a covalent (*i.e.* non-polar non-charge-shift) bond (C_2_H_6_), a non-polar charge-shift bond (F_2_), an ionic (*i.e.* polar non-charge-shift) bond (LiF), and a polar charge-shift bond (CH_3_OH). The color scale of the reduced potentials ranges from blue (low values) to yellow (large values). SCPs, first-order DCPs, second-order DCPs, and other arrangements are depicted as circles, triangles, squares, and stars, respectively. Covalent arrangements, ionic arrangements, and central arrangements are depicted in red, violet, and green, respectively.

The comparison of the two-electron potentials of F_2_, CH_3_OH, C_2_H_6_, and LiF shows the qualitative manifestation of both bond character dimensions (Fig. 9). The charge-shift contribution to the bond introduces a ‘hill’ or ‘ridge’ in-between the covalent arrangements (upper left and lower right). The quantification of this contribution has already been described in this article. The bond polarity appears as the tilt along the diagonal *z*_1_ = *z*_2_ (lower left and upper right) of the two-electron potential. It can been quantified as the difference between two ionic arrangements.

The four depicted two-coordinate potentials show that these effects occur independently: they are neither mutually exclusive, nor does one strictly imply the other. Thus, there are polar charge-shift bonds and non-polar non-charge-shift bonds, but also polar non-charge-shift bonds and non-polar charge-shift bonds. Therefore, instead of speaking of a third family of bonds, we suggest to denote the charge-shift character of a bond as an additional dimension of bond characterization in addition to the covalent-ionic bond polarity. We do this, even though a quantitative analysis reveals that polarity and charge-shift character are not truly statistically independent. Instead, most bonds fall into one of three categories. First, homoatomic bonds, which all have a polarity close to zero, but can have none or considerable charge-shift strength. Second, normal ionic bonds, which have a charge-shift strength of zero with a varying polarity. And third, polar bonds, for many of which the charge-shift strength and the polarity—as defined in this article—are identical. This close relationship between polarity and charge-shift strength for a subset of bonds is intuitively comprehensible: if there is a ridge between the covalent arrangements (charge-shift character) in the two-coordinate potential, an increasing tilt along the diagonal (polarity) does necessarily also increase the prominence of the ridge.

**Fig. 10:**
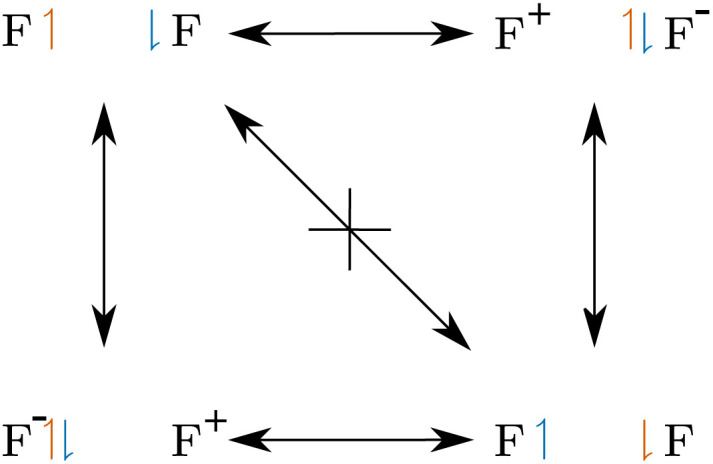
Electron exchange in F_2_. In contrast to ordinary covalent bonds, the concerted exchange is prevented by a large probability barrier.

Given this definition, one may ask what the actual physical meaning of charge-shift bonding is. We assume, that the sharing of the bond electrons is the fundamental mechanism of covalent bonding. This sharing can be investigated by analyzing the exchange of the electrons in the bond, *i.e.* the delocalization path between the two covalent arrangements (SCPs). For charge-shift bonds, like F_2_, the concerted exchange of the electrons is prevented by a large probability barrier. The exchange of the electrons is instead described by a shift of charge to one of the fluorine cores (Fig. 10). Note the resemblance of this qualitative scheme to the quantitative two-electron potential of F_2_ (Fig. 6).

## Conclusion

The concept of charge-shift bonding originates from valence bond theory and was developed to describe—among other things—the qualitative differences between the central bonds in ethane and the fluorine molecule. Yet the valence bond description relies on a covalent reference state. With probability density analysis, the many-electron probability density |*Ψ*|^2^ and its negative logarithm, the probability potential, are investigated. Most probable electron arrangements are identified as local minima of the probability potential. Each local minimum represents a corresponding basin of attraction of close electron arrangements within the probability density. A two-electron exchange on a single bond is then described as a path connecting two of these local minima. Distinct types of exchange paths are found: one describing a concerted exchange of the two electrons and another describing a step-by-step exchange *via* an ionic intermediate arrangement—a shift of charge. The contribution of a path to the bond can be inferred from its probability potential barrier. The difference between the barriers of the charge-shift exchange and the concerted exchange has consequently been defined as the charge-shift strength, which is dimensionless and size-consistent. Charge-shift strengths calculated from explicitly correlated molecular orbital wave functions correlate well with the charge-shift resonance energies from valence bond theory thus supporting the charge-shift concept independent of valence bond theory. It should be emphasized, that the charge-shift strength is independent of the bond polarity in the sense, that they are neither mutually exclusive nor does one strictly imply the other. It is therefore suggested to characterize bonds by both their polarity and their charge-shift strength.

## Methods

### Computational approach

All geometries were taken from the NIST Computational Chemistry Comparison and Benchmark Database.^24^ All PDA calculations were done with Slater-Jastrow wave functions. These are compact correlated wave functions used in the quantum Monte Carlo community. Mean field wave functions such as Hartree–Fock are unsuitable for this analysis due to unreasonably high ionic contributions to the bond. For the Slater part, two ansatzes have been employed: a single determinant of Kohn–Sham orbitals from PBE0 ^25^ calculations (for Fig. 8) and linear combinations of Slater determinants from complete active space self-consistent field (CASSCF(2,2)) calculations (all other figures). The Molpro^26^ package was used for all CASSCF and ORCA^27–29^ for all PBE0 calculations. For all-electron calculations, the Slater-type TZPae basis^30^ was used, while the BFD pseudopotential with the corresponding VTZ basis by Burkatzki *et al.*^31^ was used for valence-only calculations. The all-electron basis was used for all elements up to the first period, while for heavier atoms the valence-only basis was used. For the use of the TZPae basis in Molpro, each Slater function was expanded into 14 primitive Gaussian functions.^32,33^ A generic sm666 Jastrow factor^34,35^ was added to the wave functions with subsequent optimization of all parameters (including configuration interaction and orbital coefficients). All charge-shift resonance energies were calculated with breathing-orbital valence bond^36^ at the BOVB(2,2)/cc-pVDZ level with XMVB.^37,38^

The PDA was performed with Amolqc^39^ and inPsights.^40^ The Newton method and gradient norm minimization with L-BFGS^41^ were used to identify saddle points. In heteroatomic bonds, often only one ionic path is found, and no central DCP can be identified. In this case, the central barrier is calculated at the point of locally minimal slope on the ridge between the two covalent minima. If this does not exist either, the point of locally minimal curvature is taken. Likewise, if covalent minima (SCPs) cannot be identified, the points of locally minimal slope in the respective valleys are used to calculate the barriers. A rationale for and detailed description of this procedure is laid out in the ESI (ESI Fig. 1).†

## Data availability

The data that support the findings of this study are openly available in the Open Science Framework at https://osf.io/4tn37/.^42^

## Author contributions

Conceptualization, M. V. H., L. R., A. L.; data curation, M. V. H.; formal analysis, resources, A. L.; software, M. V. H., L. R., A. L.; supervision, A. L.; visualization, M. V. H., L. R.; writing – original draft, M. V. H., L. R. writing – review & editing, M. V. H., L. R., A. L. All authors have read and agreed to the published version of the manuscript.

## Conflicts of interest

There are no conflicts to declare.

## Supplementary Material

SC-015-D4SC01674B-s001
