# Supplementary material for: Identifying a real space measure of charge-shift bonding with probability density analysis
Source: Chem Sci. 2024 May 13;15(23):8820–7. doi: 10.1039/d4sc01674b (PMC11168100; doi:10.1039/d4sc01674b)
Supplement: SC-015-D4SC01674B-s001 [file SC-015-D4SC01674B-s001.pdf]

Electronic Supplementary Material (ESI) for Chemical Science.
This journal is © The Royal Society of Chemistry 2024

Identifying a Real Space Measure of Charge-Shift Bonding with Probability Density Analysis†

Michel V. Heinz,‡a Leonard Reuter,‡a and Arne Lüchow*a

Electronic Supplementary Information

Algorithm for determination of the charge-shift strength

In the case of homoatomic bonds, the structure and delocalization critical points can easily be identified by different stationary points of the two-electron potential. However, for heteroatomic or polar bonds, critical points can change or vanish, due to the nature of topology. Thus, when determining the central barrier and no central DCP can be identified, the point of minimal slope along the ridge (inflection point) between the two covalent minima is used. In cases, where the point of minimal slope also vanishes the point of minimal curvature is taken. This results in a continuous definition for the central barrier, that can be easily extended (see Supplementary Fig. 1).

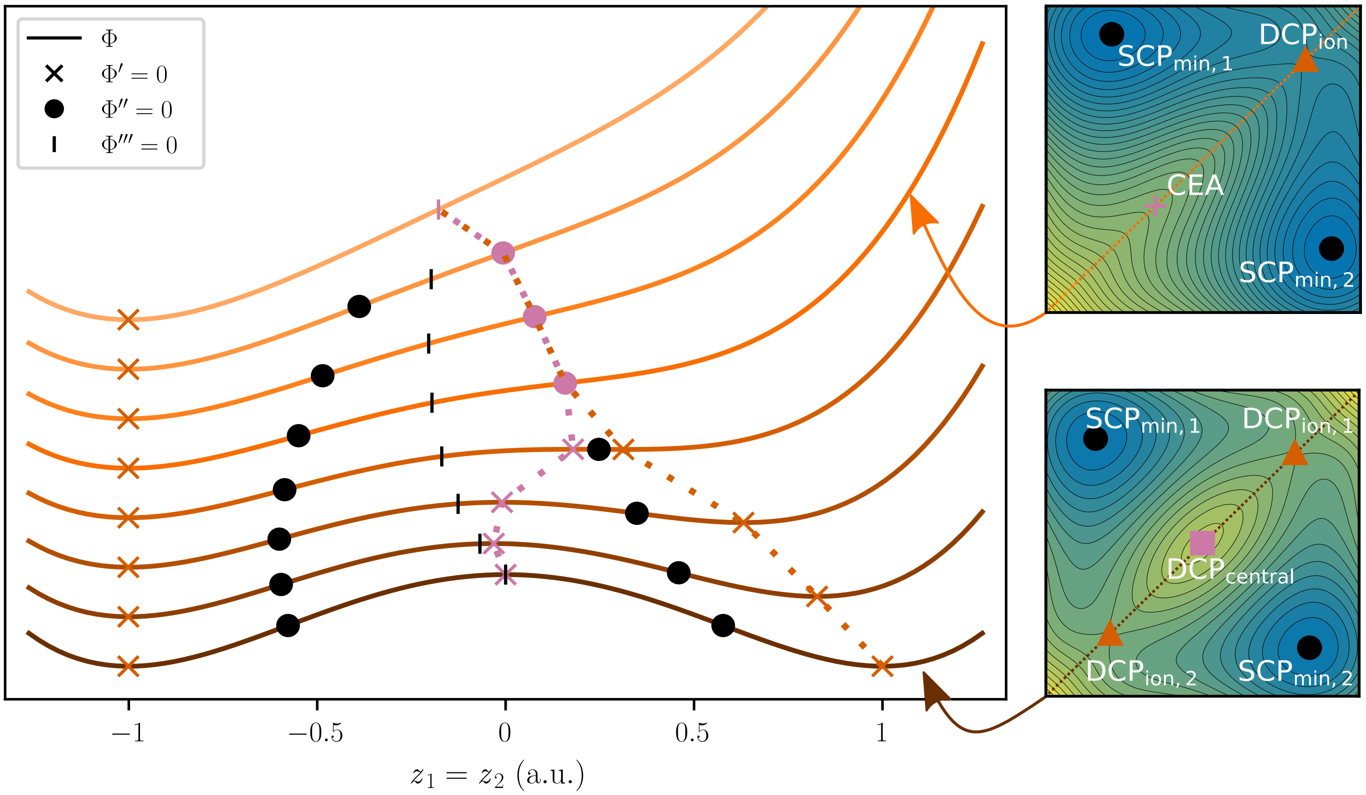

Supplementary Fig. 1 Plot of the probability potentials on the ridge of the two-electron potential (here for the line $z_1 = z_2$) for a model system. The lowest curve represents a homoatomic bond (brown dotted line in the lower right figure), while all other curves represent heteroatomic bonds with increasing polarity (one shown as orange dotted line in the upper right figure). The points marked in pink denote the continuous definition of the central electron arrangement (CEA) for different polarities.

To determine the critical points on this ridge, the values for the probability potential are evaluated and fitted with an nth degree polynomial (where n depends on the number of sample points). Through differentiation, the critical points are identified and the values for the central barrier can be recorded. Analogously, the covalent minimum can disappear. Again, a covalent arrangement is identified in its place by determining the point of smallest slope in the resulting valley.

Wave function dependency

All results were derived from a CASSCF(2,2) wave function with a Jastrow correlation function[1,2] except for Fig. 8, where a single determinant of PBE0 Kohn Sham orbitals has been used instead of the CASSCF(2,2) part. This level guarantees

static and dynamic electron correlation to be taken care of for the bond electrons. This is important as many-electron properties are investigated. However, for different levels of wave functions the trends discussed in this article remain the same, while the charge-shift strength changes for each bond (Supplementary Fig. 2). The values of $\Delta_{CS}\Phi$ are largest for the very similar VBSCF(2,2) and CASSCF(2,2) function with static correlation only. The dynamical correlation with the Jastrow correlation factor diminishes $\Delta_{CS}\Phi$. The multi-determinant ansatz is necessary to obtain a meaningful description of F_2, but the values of $\Delta_{CS}\Phi$ calculated with one determinant using PBE0 orbitals fits well into the plot.
It should be noted, that the VB charge-shift resonance energy also varies with both the VB flavor and the basis set.[3,4]

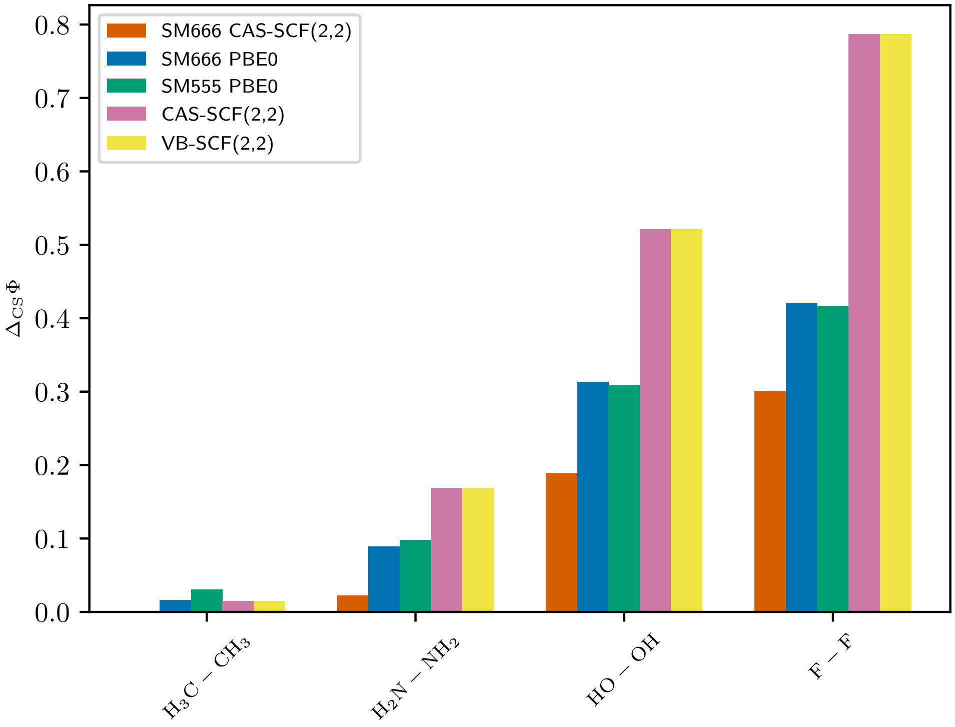

Supplementary Fig. 2 Charge-shift strength for different wave function levels computed with the TZPae basis set.[5] For the H_nX-XH_n, X = C, N, O, F, series.

Polarity Index

The polarity is described by the difference $\Delta_{POL}\Phi$ in probability potentials for the two different ionic arrangements. This difference can be understood as an average slope of the two-coordinate potential along the diagonal between the two covalent arrangements. However, in contrast to a slope, the difference is dimensionless. In homoatomic bonds, the two ionic arrangements are usually (with the exception of ethane) identified as first-order DCPs. For these systems, both ionic arrangements have the same potential, the polarity is trivially zero. In heteroatomic bonds, one ionic arrangement is usually identified as an SCP, i.e. a local minimum of the probability potential Φ. The other ionic arrangement can usually not be identified as a DCP anymore. Again, in analogy to our treatment of the central arrangement, the first order DCP can be tracked for a smooth transition of a model system from non-polar to polar bonding. It is revealed that when the first-order DCP disappears it merely merges with the central DCP, such that the two points coincide (cf. the dotted orange line in Supplementary Fig. 1). The PDA polarity index defined this way correlates well with the difference in electronegativity of the two bond atoms (Supplementary Fig. 3).

Dissociation

Another difference between the VB and PDA descriptions of charge-shift bonding, which can not be seen in Fig. 8, is the dissociation behavior: since the ionic contributions vanish for the dissociated fragments, the charge-shift resonance energy E_{CS} goes to zero as well. In contrast, with PDA, not only do both the central barrier $\Delta_C\Phi$ and the ionic barrier $\Delta_I\Phi$ increase, but their difference, the charge-shift strength $\Delta_{CS}\Phi$, increases as well. It is however physically very sensible that

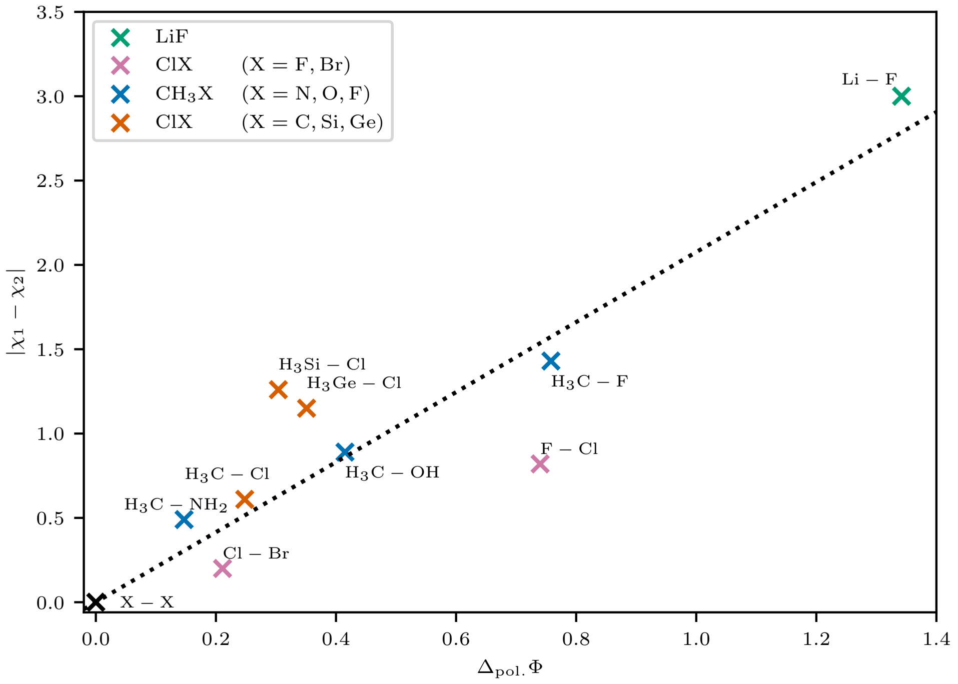

Supplementary Fig. 3 Difference of the Pauling electronegativity vs. the PDA polarity index for different heteropolar compounds. Calculated for Jastrow correlated CASSCF(2,2) wave function with the TZPae basis set.[5]

$\Delta_{CS}\Phi$ should behave this way: for a very large distance (e.g. 10 angstroms), any exchange of electrons should be really unlikely. But if there is any exchange, it is even less unlikely, that both electrons leave their fragments and travel the 10 angstroms at the same time. After all, the probabilistic measure, which we define and investigate is different from any energetic measure.

References

1 A. Lüchow, A. Sturm, C. Schulte and K. Haghighi Mood, *The Journal of Chemical Physics*, 2015, **142**, 084111.

2 A. D. Güçlü, G. S. Jeon, C. J. Umrigar and J. K. Jain, *Physical Review B*, 2005, **72**, 205327.

3 L. Zhang, F. Ying, W. Wu, P. C. Hiberty and S. Shaik, *Chemistry - A European Journal*, 2009, **15**, 2979–2989.

4 S. Shaik, D. Danovich, B. Silvi, D. L. Lauvergnat and P. C. Hiberty, *Chemistry - A European Journal*, 2005, **11**, 6358–6371.

5 E. van Lenthe and E. J. Baerends, *Journal of Computational Chemistry*, 2003, **24**, 1142–1156.
